# Green microwave quantum dots as luminescent probes for quantifying prucalopride: consistency of content and application to pharmacokinetic studies

**DOI:** 10.1186/s13065-023-01002-4

**Published:** 2023-07-19

**Authors:** Baher I. Salman

**Affiliations:** Pharmaceutical Analytical Chemistry Department, Faculty of Pharmacy, Al-Azhar University, Assiut Branch, Assiut, 71524 Egypt

**Keywords:** Prucalopride, N-CQDs, Fluorescence, Human plasma, Content uniformity

## Abstract

Prucalopride (PCP) is a medication used for the management of constipation via regulating bowel motions. PCP is widely used all over the world. So, novel, rapid, and highly sensitive carbon dots N-CQDs were obtained from *Eruca Sativa* juice via microwave approach in 4 min. The luminescence power of N-CQDs was declined by the increasing prucalopride concentration at emission 518 nm with linearity ranged from 3.00 to 200.00 ng mL^−1^. The luminescent antecedent was utilized for the test of PCP in human plasma with the rate of recovery extending from 95.06 to 98.40%. The new technique is an eco-friendly analytical method that can be easily applied in clinical laboratories. This assay is also simple, sensitive, and applied to therapeutic laboratories and subsequent pharmacokinetic studies in several clinical laboratories. Furthermore, the N-CQDs nano-sensor was able to distinguish the target drug from interferents commonly found in human plasma, indicating its high specificity and selectivity for PCP detection.

**Supplementary Information:**

The online version contains supplementary material available at 10.1186/s13065-023-01002-4.

## Introduction

Prucalopride (PCP) Additional file 1: Fig. S1 is the first novel chemical class of dihydro benzofurancarboxamide derivatives. PCP is applied for the management of constipation by improving colon motility [1, 2]. Worldwide, chronic constipation is a frequent condition, the estimated population prevalence varies by geography, from 8.75% in the Asia–Pacific to 27% in Western nations [3].

One spectrofluorimetric approach has been published for the assay of PCP [4] which is based on the analysis of PCP using complexation between PCP and terbium (Tb^+3^) in the presence of 8-hydroxyquinoline and phosphate [4].

The reported spectrofluorimetric method [4] has varying drawbacks as using expensive elements such as terbium chloride and using different chemicals such as 8 hydroxyquinoline which increases environmental pollution and lacks biological applications.

Few methods have been reported for estimation of PCP as HPLC [5, 6], spectrophotometric [7], and electrochemistry [8].

The proposed technique is the new creative method for quantifying PCP with various applications as human plasma (pharmacokinetic study), content uniformity, and pharmaceutical dosage form with high sensitivity other than reported methods [4–7].

Intelligent nanomaterials called carbon dots have different applications in multiple domains. These types of quantum dots have applications in bioimaging, sensing, and optoelectronics due to their tunable size, and high photostability [9–11].

Microwave amalgamation [12, 13] of the quantum dots may be a novel method recently practiced decreasing the amalgamation time from hours to minutes and accomplishing green amalgamation with high quantum yield items [12, 13].

Green nitrogen-doped quantum dots and environmentally friendly sources are currently superior candidates for the creation of luminous dots. These quantum dots have a unique structure, where the nitrogen was doped in the carbon skeleton. This doping creates energy states within the bandgap of carbon, which can be excited to generate green fluorescence measured at 518 nm [12, 13].

Various nanomaterials were reported from different sources as glutathione, polyvinyl pyrrolidine, Cadmium sulfide dots, and selenium nanoparticles [14]. The reported nanomaterials have a size range (20.0 to 123.5 nm) which is large compared with the presented work (2.1 nm).

Eruca sativa is a very inexpensive plant that is generally grown in many countries. Eruca Sativa contains various active ingredients such as vitamins, riboflavin, sugars, folic acid, fiber, vitamin A, and vitamin B1 [15, 16] which were used for producing nitrogen-doped quantum dots with various functional groups [13].

This study aims to synthesize novel innovations of green emission N-CQDs as a luminescence sensor which has shown promising results in the detection of PCP with low coasting.

## Methodology section

### Materials and chemicals

Prucalopride (PCP, purity 99.96%) was provided from Marcyl Pharmaceutical Industries, Egypt. Prucasoft® 2 mg (batch No. 2031461) was provided from Marcyl Pharmaceutical Industries, Egypt.

The PCP standard solution was performed and prepared using ultrapure water (100 µg mL^−1^).

### Instruments of the analytical method

Various analytical equipment were utilized in this study [13] FS5 spectrofluorometer (UK), with Fluoracle® software. Zetasizer (UK) [13]. Microwave (MED Future). Transmission electron microscopy [13]. Fourier Transform Infrared (FTIR) Germany. Powder X-ray diffraction (PXRD) (Philips) [13].

### Preparation of nitrogen carbon dots

The N-CQDs were prepared using a pyrolytic strategy as reported in [13]. Forty milliliters of plant juice were relocated to the microwave flask and then implanted in a microwave oven for 4 min [13]. The formed component was suspended in 50 mL ultrapure water with sonication (30 min) followed by centrifugation for 10 min (4000 rpm). The clear solution was filtered via a 0.45 μm cellulose membrane. The resulting yellow filtrate-colored solution was used for experiments [13].

### The fluorimetric procedure of PCP

Mix 0.8 mL of N-CQDs with 1.0 mL of BR buffer (pH 6.2) into a 5 mL calibrated flask, after that 1.0 mL of PCP was added to get the final range (3.00–200.00 ng mL^−1^), mixed thoroughly, and the volume replenished with ultrapure distilled water. RFI was measured at λem 518 nm (excitation 450 nm) after 8 min.

### Analysis of PCP in commercial dosage form

Prucasoft® tablets (10 tablets), each containing 2.0 mg, were measured, crushed, and properly combined [4]. After that, an amount equal to 10.0 mg of PCP was added to a calibrated flask containing 50 mL of distilled water. To obtain a concentration of 100 µg mL^−1^, the sonicated was carried out for 20 min, filtered, and then diluted to a volume of 100 mL using double-distilled water [4, 13].

Each tablet of the Prucasoft® medicine was individually weighed and then pulverized for the content uniformity test [13, 17, 18]. Each tablet was prepared using a commercial dosage form procedure.

### Application in human plasma

An amount of PCP solution was mixed with 500 μL of plasma in a centrifuge tube and 1.0 mL of acetonitrile was used as a protein precipitant [19–21]. The solution was vortexed for 30s [13] and then adjusted to 10 mL to get the concentration range. Centrifuge this mixture at 3500 rpm for 30 min, then 1.0 mL of the clear solution was performed in the analytical methodology to obtain a final concentration between 3.00 and 200.00 ng mL^−1^.

### Pharmacokinetic Study of PCP

This study was performed depending on the ethical committee of Al-Azhar University with approval No. ZA-AS/PH/8/C/2023, and informed assent was gotten for any tests with humans.

Five healthy human volunteers received one oral dosage of PCP tablets (2.0 mg/tablet). Three milliliters of the blood specimen were obtained intravenously into heparinized tubes at time intervals of 0.25, 0.5 to 30 h) [22]. To separate the plasma, the blood specimen was centrifuged for 30 min (5000 rpm) [13]. One milliliter of the human plasma samples was centrifuged with 1.0 acetonitrile as a protein precipitant at 3500 rpm for 30 min [13, 19–21].

## Results and discussion

Intelligent nanomaterials called carbon dots have different applications in multiple domains. These types of quantum dots have applications in bioimaging, sensing, and optoelectronics due to their tunable size, and high photostability [12, 14, 23, 24]. The creative method was easily utilized for the quantifying of PCP in human plasma and commercial products.

### Surface morphology of N-CQDs

**Fig. 1:**
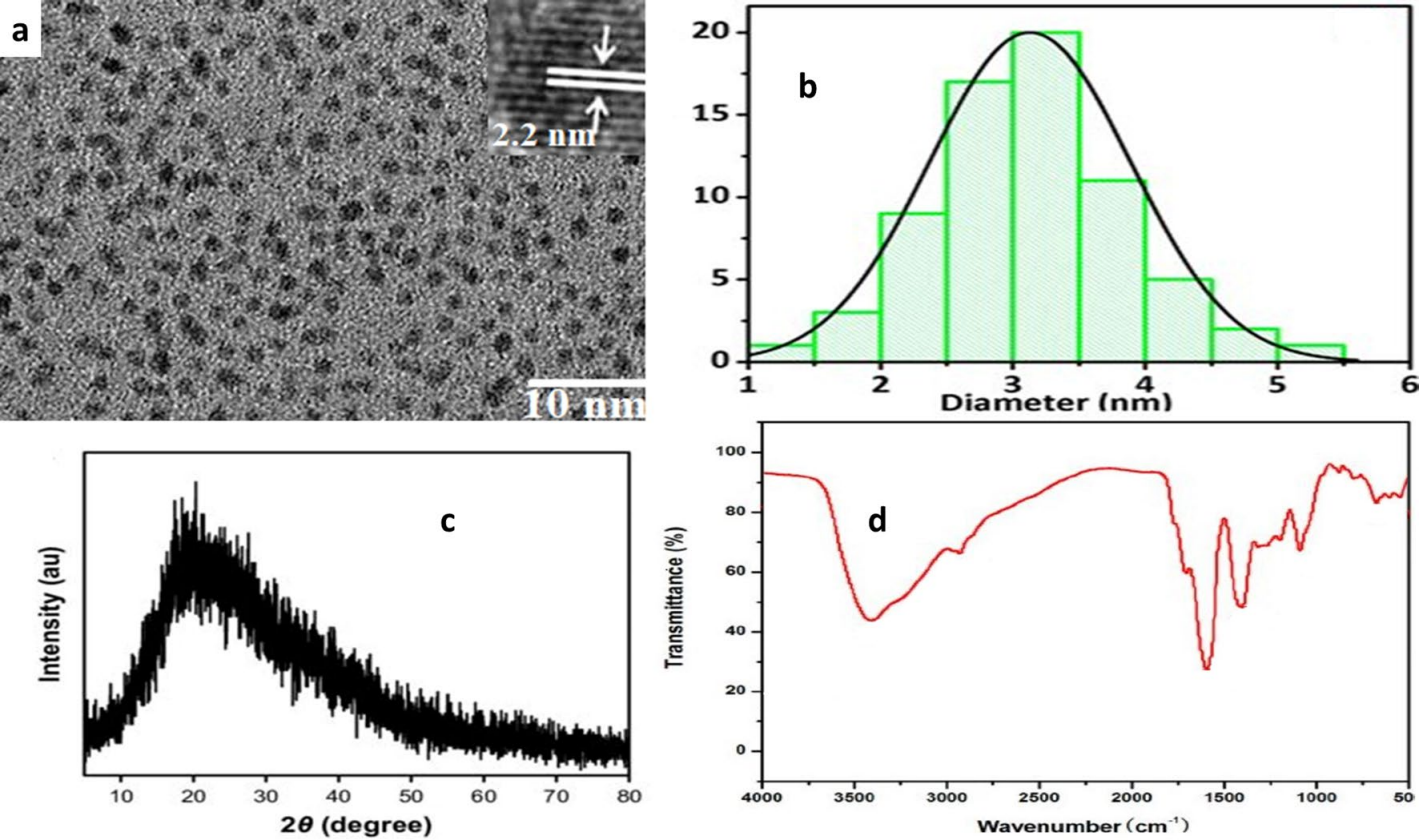
Morphological characters of N-CQDs, **a** TEM image for N-CQDs **b** DLS image of carbon dots, **c** PXRD for N-CQDs **c** and **d** FTIR spectroscopy for examination of N-CQDs

The average size diameter of N-CQDs was performed via transmission electron microscopy (TEM) [13], and it was found the diameter was equal to 2.2 nm ± 0.21, as in Fig. 1a.

Additionally, the size diameter was confirmed using DLS. The diameter was found 3.0 nm, as shown in Fig. 1b. This result is refer to the smaller particle size of N-CQDs compared to other published methods [9, 11, 18].

Furthermore, PXRD was used to examine N-CQDs synthesis, a characteristic peak was attended at 24.60 °C, consistent with other reported methods [9, 11, 13] as seen in Fig. 1c.

FTIR spectroscopy was conducted for functional groups examinations. As shown in Fig. 1d characteristic peaks at 3400 and 2930 cm^−1^ related to (−NH, −OH) and 2900 cm^−1^ for −CH [13]. In addition, peaks at 1600 and 1450 cm^−1^ refer to (−C = O and −C-N) [13]. Additionally, the peaks at 1100 and 680 cm^−1^ are associated with (N-O and C-O), respectively Fig. 1d.

**Fig. 2:**
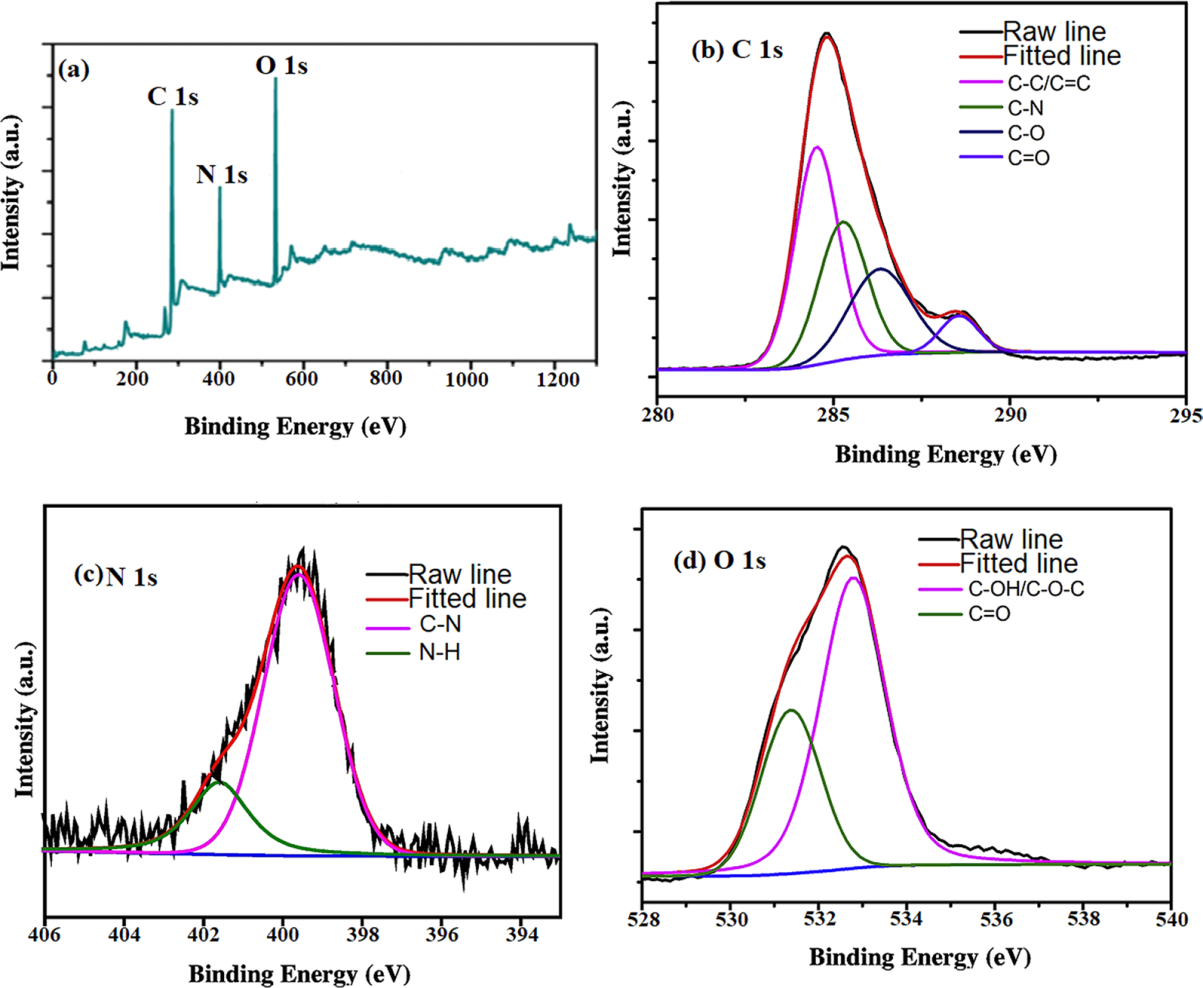
**a** XPS spectrum, **b** C 1 s spectrum **c** N 1 s spectrum and **d** O 1 s spectrum for element analysis of N-CQDs

Two instruments were carried out for elemental analysis, EDX was carried out at the first as shown in Additional file 1: Fig. S2 [13]. Secondly, XPS was performed for N-CQDs. Three distinctive peaks at (284.9, 395.6, and 538.5 eV), interrelated to C1s, N1s, and O1s, respectively [13]. This means that the carbon dots are mainly made up of O (43.0%), C (37.89%), and N (19.11%)[25] Fig. 2a.

The surface passivation of carbon dot particle surfaces is what causes the creation of N (19.11%). Four characteristic peaks were seen in the C1s curves (Fig. 2b) related to C = C, C-N, C-O, and C = O groups [13, 25]. The C-OH, C-O-C, and C = O spectra for O1s curves had two peaks (Fig. 2c) [9, 13, 26]. The C-N and N-H causes the N1s spectra to show two peaks as illustrated in Fig. 2d [13, 27].

Single-point approach [13] was used to investigate the QY of N-CQDs.

$${{\varvec{Q}}}_{{\varvec{N}}{\varvec{C}}{\varvec{Q}}{\varvec{D}}{\varvec{s}}}={{\varvec{Q}}}_{{\varvec{Q}}{\varvec{u}}{\varvec{i}}{\varvec{n}}{\varvec{i}}{\varvec{n}} }\times \frac{{{\varvec{F}}}_{{\varvec{N}}{\varvec{C}}{\varvec{Q}}{\varvec{D}}{\varvec{s}}}}{{{\varvec{F}}}_{{\varvec{Q}}{\varvec{u}}{\varvec{i}}{\varvec{n}}{\varvec{i}}{\varvec{n}}}} \times \frac{{{\varvec{A}}}_{{\varvec{s}}{\varvec{t}}}}{{{\varvec{A}}}_{{\varvec{N}}{\varvec{C}}{\varvec{Q}}{\varvec{D}}{\varvec{s}}}} \times \frac{{{\varvec{\eta}}}^{2} ({\varvec{N}}{\varvec{C}}{\varvec{Q}}{\varvec{D}}{\varvec{s}})}{ {{\varvec{\eta}}}^{2} ({\varvec{Q}}{\varvec{u}}{\varvec{i}}{\varvec{n}}{\varvec{i}}{\varvec{n}})}.$$

The quantum yield was observed 41.39% [13].

### The optical studies of N-CQDs

Additional file 1: Fig. S3 displays two characteristic peaks at 229 and 296 nm which agree with other reported method [13]. Additionally, N-CQDs generate the fluorescence peak at 518 nm (excitation at 450 nm) as in Additional file 1: Fig. S3.

**Fig. 3:**
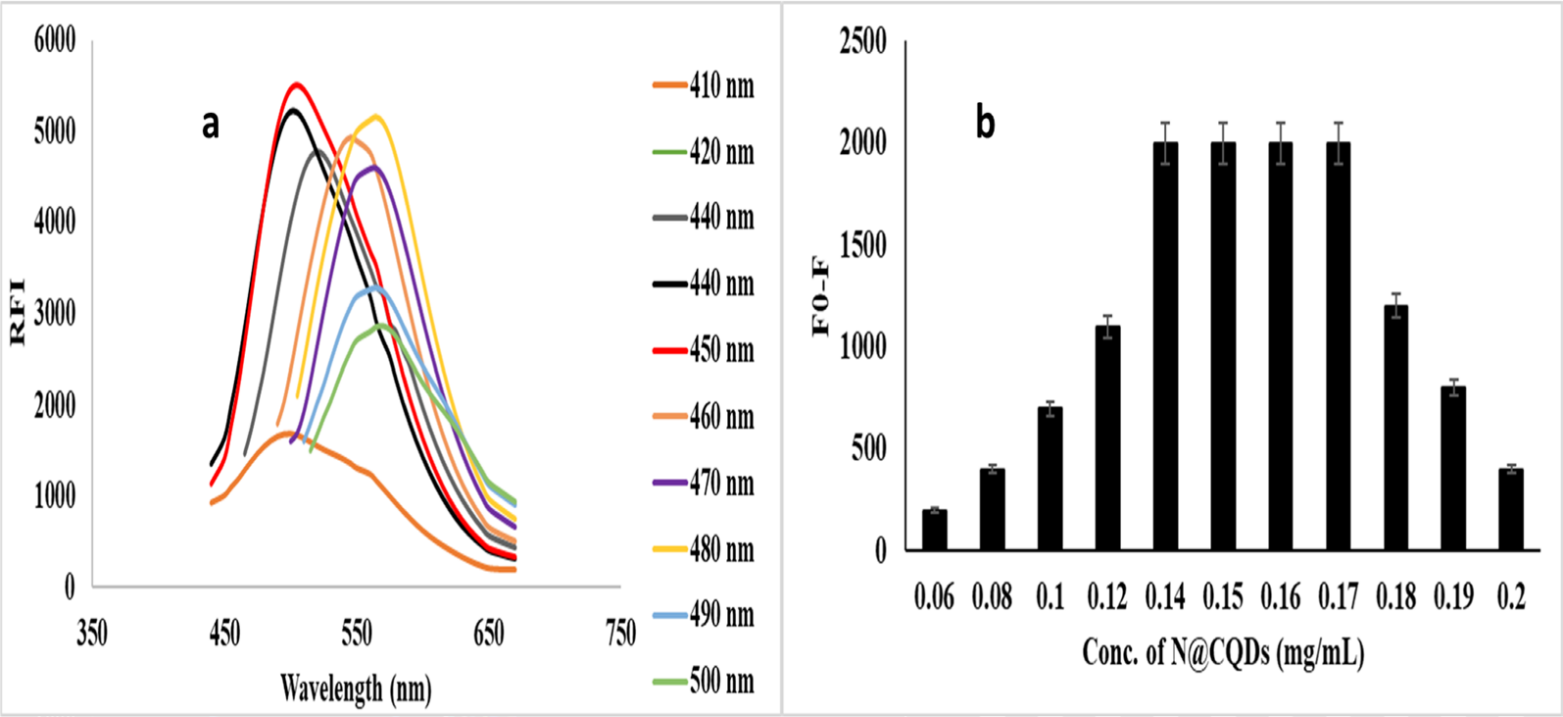
**a** Excitation-dependent emission spectra, and **b** Effect of N-CQDs concentration on the reaction with PCP (100 ng mL^−1^)

The excitation-dependent emission study was tested at wavelengths from 390 to 470 nm. It was reported a red shift in the emission spectra of N-CQ dots with decreasing the fluorescence intensity, demonstrates the excitation-dependent emission of carbon dots [13, 18] Fig. 3a.

Additionally, it investigated how the temperature affected the fluorescence intensity of N-CQDs. Temperatures above 25 °C degrees resulted in a decrease in fluorescence intensity [9, 11, 13].

To study the photostability of quantum dots, N-CQDs were exposed to UV light for varying lengths of time, ranging from 1 to 100 min. After 100 min of illumination, the particles showed great stability and did not affect RFI Additional file 1: Fig. S4.

### The optimization of analytical procedure

The impact of pH on fluorescence was studied in the presence and nonattendance of PCP. The existence of numerous functional groups in N-CQDs is watched to be relentlessly extinguished within the pH extent of 6–6.8, with an unsteady diminish in RFI with expanding pH to 6.8. Hence, the perfect pH of 6.2 was chosen Additional file 1: Fig. S5.

As seen in Fig. 3b, the effect of concentration of quantum dots in the reaction with PCP. It was obtained that 0.15 mg mL^−1^ is the most stable quenching observed.

Response times were examined at different time intervals from 0 to 30 min. The quenching response was detected after 8 min Additional file 1: Fig. S6.

### The validation of the created approach

**Fig. 4:**
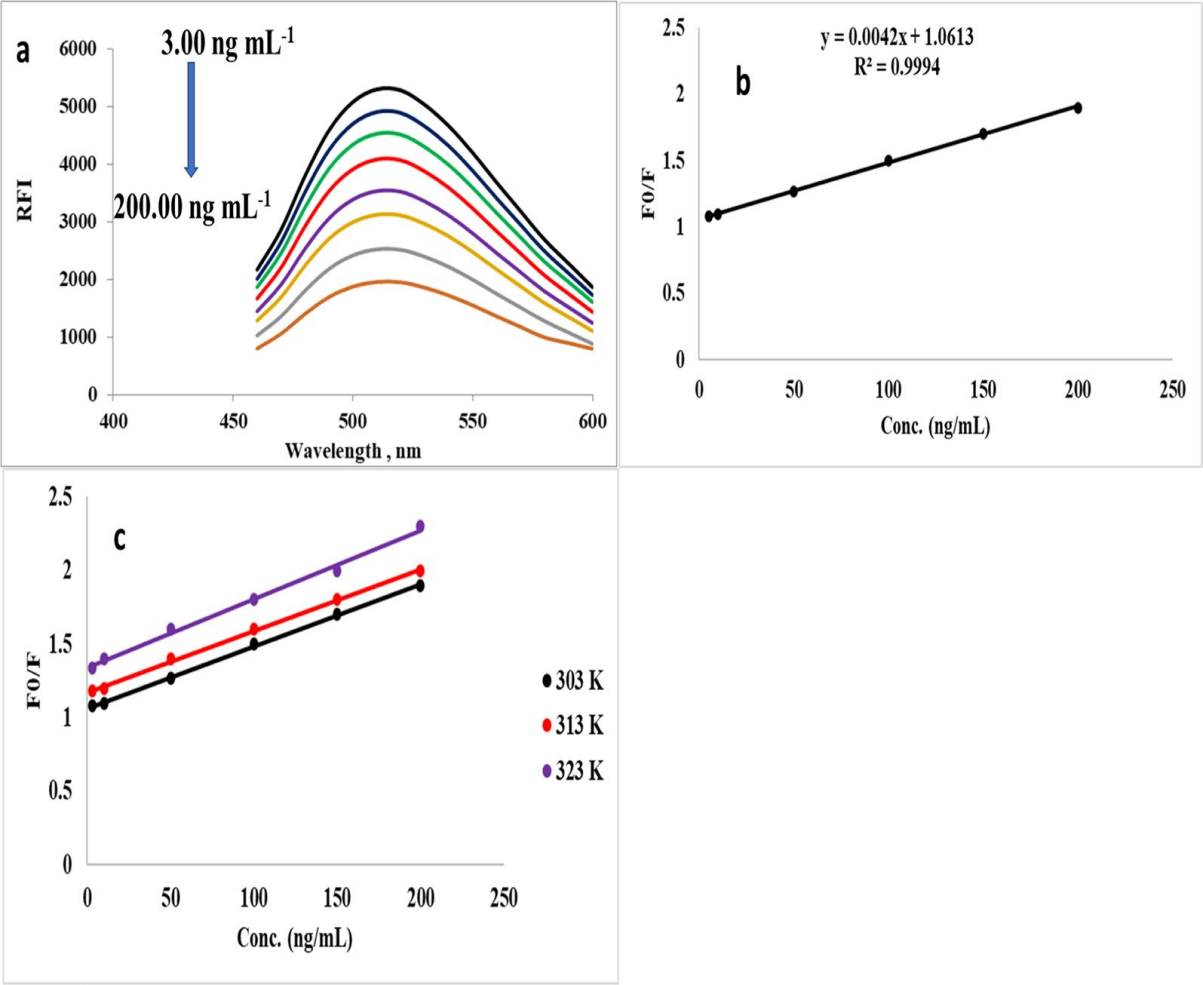
**a** Reaction between N-CQDs with various concentration of PCP **b** Stern–Volmer equation between Quantum dots and PCP and **c** Stern–Volmer plots for the quenching of the fluorescence of N-CQDs at three different temperature settings (303, 313, and 323K) by different concentrations of PCP

The created technique was validated using US-FDA and ICH rules [28, 29]. N-CQD fluorescence declined at 518 nm with the increasing of PCP concentration Fig. 4a.

The linearity was performed with the Stern–Volmer model [13], and excellent linearity was performed 3.00 – 200.00 ng mL^−1^ range Fig. 4b.

$$ {\text{F}}_{0} /{\text{F }} = { 1} + {\text{Ksv }}\left[ {\text{Q}} \right] $$

The crated technique is a sensitive method with LOD equal to 0.78 ng mL^−1^ and LOQ equal to 2.38 ng mL^−1^, as shown in Table 1.

**Table 1:** Statistical parameters for analysis of PCP using N-CQDs

Parameter	PCP
λ_ex_ (nm)	445
λ_em_(nm)	518
Concentration range (ng mL^−1^)	3.0–200.0
Determination coefficient (r^2^)	0.9994
Slope	0.0042
Intercept	1.061
SD the intercept (Sa)	0.001
LOD (ng mL^−1^)	0.78
LOQ (ng mL^−1^)	2.38

A range of PCP concentrations (3.0, 50.0, 100.0, 150.0, and 200.0 ng mL^−1^) was carried out to examine the accuracy. As shown in Table 2, the %RSD values were found from 0.34 to 1.41, with a high percentage of recovery (99.98 to 102.72%).

**Table 2:** The accuracy, intra-day precision and inter-day precision validations of the creative approach

Sample number	Taken (ng mL^−1^)	Found (ng mL^−1^)	% Recovery^*^ ± RSD
1	3.0	3.01	100.33 ± 0.60
2	50.0	50.11	100.22 ± 0.82
3	100.0	99.98	99.98 ± 0.34
4	150.0	152.28	101.52 ± 1.04
5	200.0	205.44	102.72 ± 1.41
Intra-day precision	50.0	50.23	100.46 ± 0.53
	100.0	100.12	100.12 ± 0.66
	150.0	151.20	100. 80 ± 0.61
Inter-day precision	50.0	49.49	98.98 ± 0.85
	100.0	99.95	99.95 ± 1.38
	150.0	150.13	100.08 ± 0.90

*Average of three determinations. *RSD* Relative standard deviation

In expansion, the intraday precision of the proposed strategy was tried in three continuous estimations at three levels (50, 100, and 150 ng mL^−1^). On the other hand, inter-day precision was inspected utilizing 3 concentrations measured in triplicate on 3 sequential days. The results are refer to great reproducibility and direct accuracy, with (% RSD) extending from 0.53 to 1.38% (Table 2).

The robustness of the recommended technique was examined by slight variations in analytical process conditions such as pH, buffer volume, reaction time, and quantum dot volume. Small changes in the studied parameters did not significantly affect the performance of the method Table 3.

**Table 3:** The robustness study for estimation of PCP with N-CQDs

Variations	PCP (100 ng mL^-1^)
	% Recovery^a^ ± RSD
Optimum condition	101.55 ± 0.94
1- Value of pH (BR buffer)	
6	99.71 ± 1.06
6.4	99.60 ± 0.74
2- Volume of buffer (mL)	
0.75	99.79 ± 0.50
1.25	99.30 ± 0.91
3- N@CQDs concentration (mg mL^-1^)	
0.14	99.75 ± 0.44
0.16	99.94 ± 0.92
4- Reaction time (min)	
6	99.91 ± 0.59
10	99.90 ± 0.31

^a^Mean of three determinations

The impact of the matrix was vital to assess the interference from plasma with PCP utilizing three quality control levels [13, 18, 19] for the explored drug (10.0, 100.0, and 200.0 ng mL^−1^). The rate of recuperation ± RSD was found to be between 96.00 ± 1.58 and 98.00 ± 1.79. The comes about shows the nonattendance of obstructions from the lattice impact with examined sedate and alludes to the tall selectivity of the proposed strategy as appeared in Additional file 1: Table S1.

Furthermore, the stability of PCP in plasma was tested at different parameters [13, 18]. The results demonstrate that PCP is stable in the plasma as in Table 4.

**Table 4:** Stability of PCP in plasma samples

Conditions			
Concentrations	LQC 10 ng mL^−1^	MQC 100 ng mL^−1^	HQC 200 ng mL^−1^
Three Freeze–thaw cycle stability (−24 °C)	96.10 ± 2.05	95. 74 ± 1.44	96.10 ± 0.84
Long-term stability (1 months at −24 °C)	97.43 ± 1.51	96.55 ± 1.81	97.00 ± 1.13
Short-term stability (12 h at −24 °C)	95.50 ± 1.28	95.48 ± 1.90	95.31 ± 1.57
Post-preparative stability (6 h at room temperature 25 °C)	97.26 ± 1.44	97.00 ± 1.11	97.33 ± 1.22
Post-preparative stability (12 h at room temperature 25 °C)	96.99 ± 2.11	97.10 ± 1.52	96.00 ± 0.89

Average of five determinations

To check the precision and accuracy of the plasma tests, the ISR of the gotten tests was performed. Concurring to Additional file 1: Table S2, the run of values between the initial and gotten tests was 2.55–5.90%.

The dilution integrity (DI) was surveyed by weakening high-concentration plasma tests inside the calibration extend of the strategy beneath examination. The comes about deviation coefficients of variety (CV%) were between 1.50 and 2.44 and recuperations between 96.80 and 98.44%. The results are refer to high precision and great accuracy limits. In this way, plasma tests with concentrations over the upper constraint of the quantitation LOQ can be dependably analyzed at suitable dilutions.

Besides, the extraction recovery was studied using various types of precipitating agents such as (methanol, ethanol, and acetonitrile). It was found the maximum recovery range was obtained using acetonitrile solvent Additional file 1: Table S3.

To rule out excipient interference, the technique's selectivity was put to the test [13]. The results show that the tablet component did not affect the results Additional file 1: Table S4.

### The reaction mechanism of the creative approach

The stern–Volmer equation was utilized to perform the reaction mechanism of the suggested approach: ${\text{F}}_{0} /{\text{F }} = { 1} + {\text{Ksv }}\left[ {\text{Q}} \right]$.

It was found the quenching impact was shown by the Stern–Volmer linearity Fig. 4b. PCP interact with the N-CQDs, coming about in QD energy/electron transfer and fluorescence rot. The Stern–Volmer band impeccably portrays this response. dynamic quenching was performed utilizing a temperature-dependent effect. The Stern–Volmer plots gotten by changing the temperatures (Fig. 4c) refer to dynamic quenching with an increment in temperature [13, 30, 31].

The Ksv values at different temperatures were calculated, and it was found Ksv was equal to 0.002, 0.006, and 0.009 for 303 K, 313 K, and 323 K respectively. The results confirm the dynamic quenching effect.

The presence of numerous functional groups on PCP not only enables the formation of hydrogen bonding interaction with the electrostatic reaction but also between PCP and N-CQDs [9, 13, 32].

### N-CQDs applications in biological samples

The proposed method was effectively used to estimate the researched medication in the human plasma due to its great sensitivity. PCP was detected in the human plasma, and the percent recovery values ranged from 95.06 ± 1.05 to 98.40 ± 1.77% when utilizing the method that was tested (Additional file 1: Table S5).

**Fig. 5:**
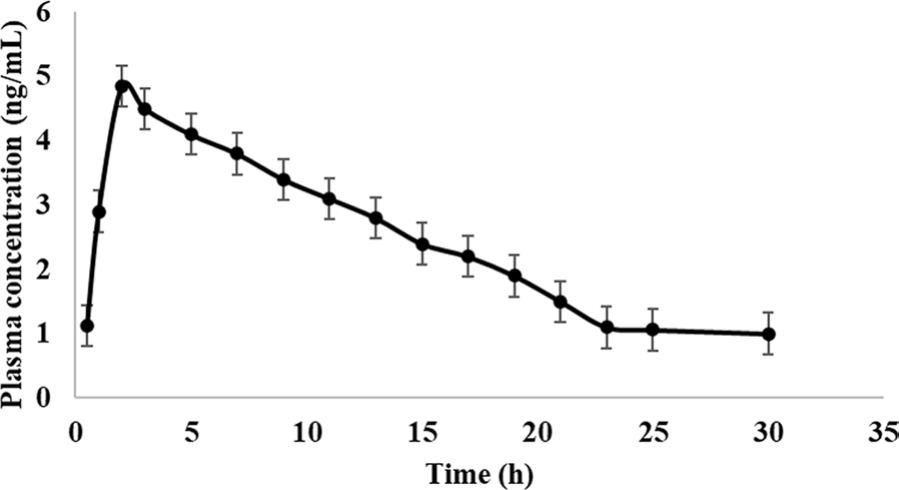
Pharmacokinetic study of PCP using the proposed fluorimetric method

Healthy human volunteers were used in the evaluation of PCP's pharmacokinetics. Using a single oral dose, the pharmacokinetic parameters were investigated (2.0 mg). C_max_ was evaluated to be 4.85 ± 0.52 ng mL^−1^, T_max_ is 2.0 ± 0.35 h, t_1/2_ was found to be 19.00 ± 0.51 h and AUC (ng·h mL^−1^) was found to be 88.60 ± 10.14 (Fig. 5) which agrees reported method [22]. The volume of distribution (V) was calculated using the following equation:

V = amount of the dose/ concentration, it was found the volume of distribution was equal to 443.

### Pharmaceutical tablets and substance consistency test

Prucalopride in pharmaceutical tablets was effectively decided to utilize the N-CQDs method, with palatable results and a great recuperation 100.88 ± 0.70. Also, the results of the t-test and F-test were found to be 1.55 and 2.31, separately [4].

To guarantee the consistency of dosing units, each unit in a clump ought to have a medicate fabric composition that falls inside a particular extent encompassing the name claim. The strategy was impeccably suited for PCP's substance consistency testing, a difficult method when utilizing conventional test strategies. This was due to the recommended method's high affectability, capacity to measure the fluorescence escalated of a single tablet extricate quickly, and high sensitivity. The think-about was carried out utilizing the USP convention [33]. The rate of recovery is shown in Table 5.

**Table 5:** Content uniformity study for assay of PCP in tablets

Dosage form No	% Labeled claim
	Prucasoft ® tablets (2 mg PCP/tab)
1	100.40
2	100.51
3	99.59
4	101.66
5	102.82
6	101.18
7	101.44
8	100.19
9	99.89
10	102.12
Mean	102.16
SD	0.66
RSD	0.64
Acceptance value (AV)*	1.58
Max. allowed AV (L1)*	15

*Acceptance value = 2.4 × SD

### Comparison to the other reported methods

In comparison to the other reported methods, which require very specialized equipment and the use of organic solvents, our suggested technique offers a straightforward procedure with little chemical consumption, no advanced procedures necessary, and reasonably high sensitivity. The results were recorded in Table 6.

**Table 6:** Comparison study between the proposed work and reported methods

Method	Linear range	Applications	References
Voltammetry	0.23 – 1.06 µg mL^−1^	Tablets	[18]
RP-HPLC	2.0 – 12.0 µg mL^−1^	Tablets	[18]
Spectrophotometry	5.0 – 60.0 µg mL^−1^	Tablets	[7]
Spectrofluorimetry	10.0 – 300.0 ng mL^−1^	Tablets	[14]
Spectrofluorimetry	3.0 – 200.0 ng mL^−1^	Tablets, human plasma, content uniformity	Our Presented study

### The greenness study of N-CQDs

The utilization of our generation of perilous squander items was diminished or halted by the application of green chemistry. The basics of green investigation can be effortlessly adjusted to expository methods in an assortment of ways, counting extraction strategies, a diminishment in natural solvents, test estimate, strategies of arrangement, utilizing eco-friendly reagents, and squander era. The adequacy of green chemistry has been compared to that of more conventional approaches employing an assortment of greenness appraisal methods (such as GAPI and AGREE) [34–36]. Explanatory strategies assessment can help in estimation the pollution that these techniques do to the environment delivered by conventional strategies [5, 6].

GAPI and AGREE were utilized for greenness evaluation for the proposed strategy [34–36]. As appeared in Table 7, the proposed strategy includes high greenness esteem compared to the detailed strategy [4]. As it were three red zones show up with plasma applications and two red zones for pharmaceutical measurement shape. Table 7

**Table 7:** Greenness evaluation of the fluorimetric approach for analysis of PCP comparing with reported method

Technique	Proposed method		Reported method
	Spectrofluorimetry		Spectrofluorimetry
Application	Plasma samples	Pharmaceutical products	Pharmaceutical products only
Organic Solvents	1.0 mL acetonitrile	Totally Free	Totally free
Range	3.0 – 200.0 ng mL^−1^		10–300 ng mL^−1^
GAPI assessment			
AGREE assessment			–

Each of the 12 parts within the clock-shaped image shown by AGREE stands for green explanatory chemistry (GAC). The recommended method's edge for the pharmaceutical dose frame is about green, as demonstrated in Table 7, but for the third AGREE run the show for off-line testing, which is unavoidable as specified within the GAPI pictogram debate. But for the third green analytical chemistry rule, which bargains with off-line testing, which must be avoided, the pictogram for human plasma is almost entirely green. The small biological effect seen within the 11th and 12th standards of AGREE assessment is due to the utilization of ACN in plasma tests earlier to examination for test arrangement. Table 7

## Conclusion

In this study, the fluorimetric technique provides an ultra-sensitive way for fast, and cheap examination of PCP using the microwave-assisted method for synthesis N-CQDs with a high quantum yield equal to 41. 39%. The technique was successfully applied in a pharmaceutical preparation with a high percentage of recovery 100.88 ± 0.70, pharmacokinetic study, and extended to assay the stability of the examined drug in plasma.

## Future plane and study limitations

N-CQDs have wide applications in pharmaceutical formulations, biomedicine, and bioimaging. These applications were considered as a limitation for these dots. The future plan is based on introducing specific functional groups to the carbon dots to be more selective and specific targeting in biomedicine.

## Supplementary Information

**Additional file 1: Fig. S1. **Chemical structure of PCP. **Fig. S2. **EDX image for N-CQDs. **Fig. S3. **Optical characters of N-CQDs. **Fig. S4.** Photostability of N-CQDs at different times. **Fig. S5.** Effect of pH range for estimation of PCP (100 ng mL^-1^) with N-CQDs. **Fig. S6.** Effect of reaction time for estimation of PCP (100 ng mL^-1^) with N-CQDs. **Table S1. **Matrix effect of the presented study for determining PCP concentration in human plasma. **Table S2. **Incurred sample reanalysis for estimation of PCP. **Table S3.** Effect of different solvents for extraction of PCP from human plasma. **Table S4. **Effect of different excipients for estimation of PCP using the proposed method. **Table S5. **Application of the proposed method for determination of PCP in spiked human plasma.

## Data Availability

The datasets used and/or analyzed during the current study are available from the corresponding author upon reasonable request.
