# Supplementary material for: Green microwave quantum dots as luminescent probes for quantifying prucalopride: consistency of content and application to pharmacokinetic studies
Source: BMC Chem. 2023 Jul 19;17(1):83. doi: 10.1186/s13065-023-01002-4 (PMC10357765; doi:10.1186/s13065-023-01002-4)
Supplement: Supplementary file 1 — Additional file 1: Fig. S1. Chemical structure of PCP. Fig. S2. EDX image for N-CQDs. Fig. S3. Optical characters of N-CQDs. Fig. S4. Photostability of N-CQDs at different times. Fig. S5. Effect of pH range for estimation of PCP (100 ng mL-1) with N-CQDs. Fig. S6. Effect of reaction time for estimation of PCP (100 ng mL-1) with N-CQDs. Table S1. Matrix effect of the presented study for determining PCP concentration in human plasma. Table S2. Incurred sample reanalysis for estimation of PCP. Table S3. Effect of different solvents for extraction of PCP from human plasma. Table S4. Effect of different excipients for estimation of PCP using the proposed method. Table S5. Application of the proposed method for determination of PCP in spiked human plasma. [file 13065_2023_1002_MOESM1_ESM.docx]

**Green microwave quantum dots as luminescent probes for quantifying prucalopride: Consistency of content and application to pharmacokinetic studies**

**Baher I. Salman**

Pharmaceutical Analytical Chemistry Department, Faculty of Pharmacy, Al-Azhar University, Assiut branch, Assiut, 71524, Egypt, [bahersalman@azhar.edu.eg](mailto:bahersalman@azhar.edu.eg), [bahersalman2013@yahoo.com](mailto:bahersalman2013@yahoo.com)


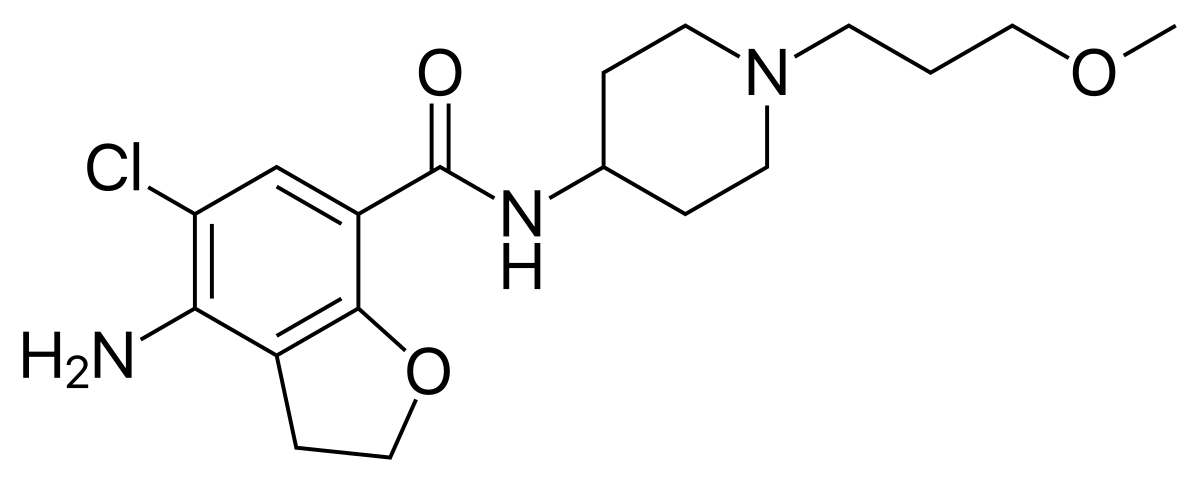


**Additional file 1: Fig. S1** Chemical structure of PCP.

**
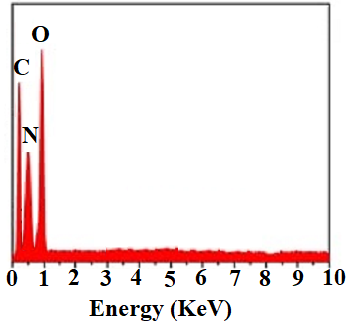
**

**Additional file 1: Fig. S2** EDX image for N-CQDs.


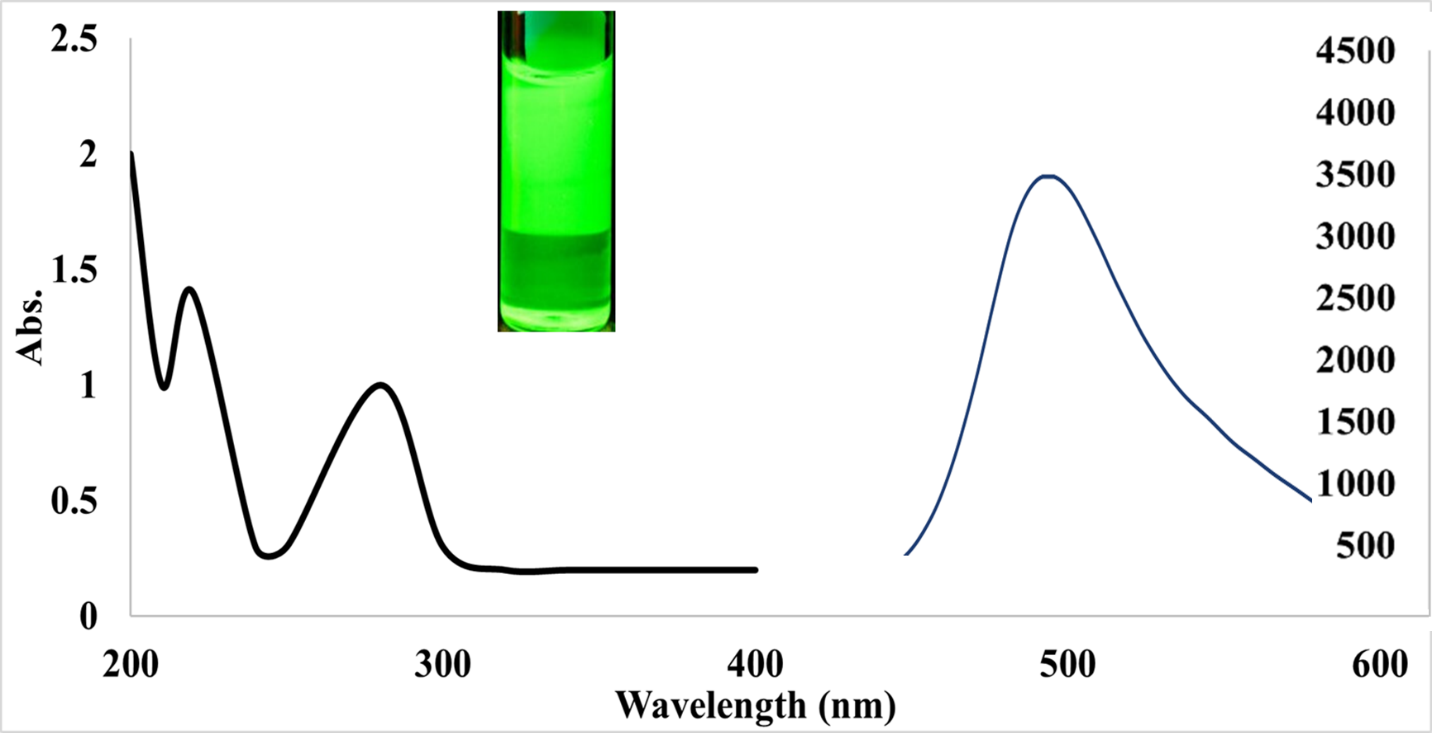


**Additional file 1: Fig. S3** Optical characters of N-CQDs.

**Additional file 1: Fig. S4** Photostability of N-CQDs at different times.


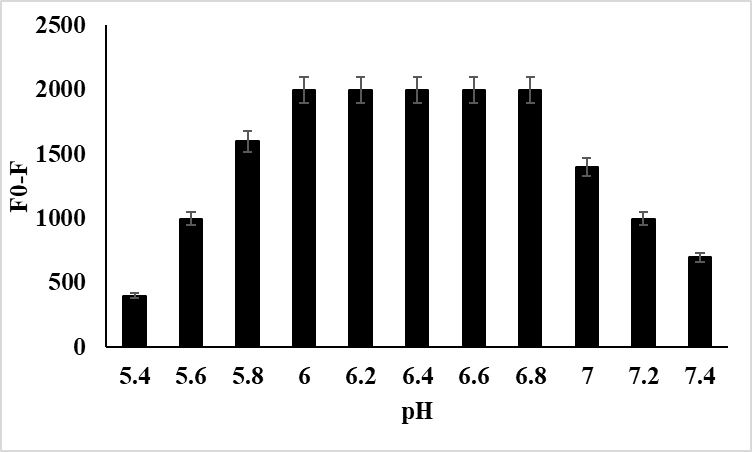


**Additional file 1: Fig. S5** Effect of pH range for estimation of PCP (100 ng mL^-1^) with N-CQDs.


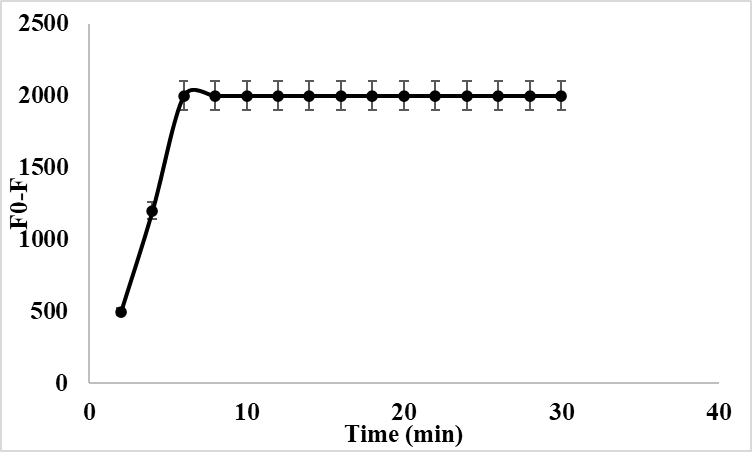


**Additional file 1: Fig. S6** Effect of reaction time for estimation of PCP (100 ng mL^-1^) with N-CQDs.

**Additional file 1: Table S1** Matrix effect of the presented study for determining PCP concentration in human plasma.

| **Inter-day assay(n=18)** | | | **Intra-day assay(n=6)** | | |  |
| --- | --- | --- | --- | --- | --- | --- |
| **Precision (CV %)** | **Accuracy (%)** | **Found**  **(ng mL^-1^)** | **Precision**  **(CV %)** | **Accuracy**  **(%)** | **Found**  **(ng mL^-1^)** | **Conc.**  **(ng mL^-1^)** |
| 1.58 | 96.00 | 0.96 | 1.81 | 97.00 | 0.97 | 10 |
| 2.08 | 96.86 | 14.53 | 1.26 | 97.93 | 14.69 | 100 |
| 1.99 | 97.32 | 24.33 | 1.79 | 98.00 | 24.50 | 200 |

**Additional file 1: Table S2** Incurred sample reanalysis for estimation of PCP.

| **Sample** | **Initial concentration* (ng mL^-1^) ± SD** | **Incurred concentration***  **(ng mL^-1^) ± SD** | **% Deviation** |
| --- | --- | --- | --- |
| **1** | 4.70 ± 0.73 | 4.42 ± 1.31 | - 5.95 |
| **2** | 4.91 ± 0.80 | 4.63 ± 1.99 | - 5.70 |
| **3** | 4.69 ± 0.66 | 4.57 ± 2.02 | - 2.55 |

*: Mean of three determinations.

**Additional file 1: Table S3** Effect of different solvents for extraction of PCP from human plasma.

| **Solvent** | **% Recovery* ± SD** |
| --- | --- |
| **Methanol** | 93.50 ± 1.80 |
| **Ethanol** | 95.40 ± 2.60 |
| **Acetonitrile** | 97.60 ± 2.33 |

*: Mean of three determinations

**Additional file 1: Table S4** Effect of different excipients for estimation of PCP using the proposed method.

|  | **Recovery* ± RSD** |
| --- | --- |
| **Mannitol** | 100.61 ± 0.33 |
| **Talc** | 100.33 ± 0.50 |
| **Starch** | 101.16 ± 0.48 |
| **Lactose** | 100.30 ± 0.29 |
| **Magnesium stearate** | 100.81 ± 0.71 |
| **Sodium chloride** | 99.80 ± 0.84 |

*: Mean of three determinations

**Additional file 1: Table S5** Application of the proposed method for determination of PCP in spiked human plasma.

| **Added conc.**  **(ng mL^-1^)** | **Found Conc.**  **(ng mL^-1^)** | | **% Recovery ^*^± RSD** |
| --- | --- | --- | --- |
| **10** | 9.60 | | 96.00 ± 1.43 |
| **50** | 48.89 | | 97.78 ± 0.84 |
| **100** | 97.72 | | 97.72 ± 1.73 |
| **150** | 147.61 | | 98.40 ± 1.77 |
| **200** | 190.12 | 95. 06 ± 1.05 | |

**^*^:** Average of six determinations.
